# 异基因造血干细胞移植治疗伴RUNX1胚系突变家族性血小板疾病伴急性髓系白血病1例报告并文献复习

**DOI:** 10.3760/cma.j.issn.0253-2727.2022.06.013

**Published:** 2022-06

**Authors:** 兰兰 王, 军 关, 平 程, 婷 张, 辉 程, 亮 邹

**Affiliations:** 武汉市第一医院血液内科，武汉 430022 Department of Hematology, Wuhan First Hospital, Wuhan 430022, China

随着基因测序技术的进步，家族性血小板疾病伴髓系恶性肿瘤（FPD/MM）的诊断率越来越高。2016年WHO在造血淋巴组织肿瘤疾病分类首次提出了胚系易感性髓系肿瘤的概念，其中大多数为遗传易感性骨髓增生异常综合征（MDS）、MDS/骨髓增殖性肿瘤（MPN）、急性髓系白血病（AML），极少部分转化为淋系肿瘤。我们应用异基因造血干细胞移植（allo-HSCT）治疗1例伴RUNX1胚系突变的FPD/AML患者，报道如下并对相关文献进行复习。

## 病例资料

患者为男性，30岁。2020年5月因“皮肤瘀点、瘀斑4个月，发热1 d”入院。患者于入院4个月前无明显诱因出现皮肤瘀点、瘀斑，未重视。既往史：2006年被诊断为急性单核细胞白血病，治疗1个疗程后未再诊治（具体不详）。本次入院体检：皮肤散在瘀点、瘀斑；血常规：WBC 4.49×10^9^/L，HGB 114 g/L，PLT 6×10^9^/L；花生四烯酸（AA）诱导血小板最大聚集率为16.5％（参考值40.0％～80.0％）；胸部CT：①右膈膨升，纵隔及心脏左移（图1）；②肝脏密度明显减低。骨髓象（图2）：增生活跃，原始细胞占35％，全片巨核细胞偶见，血小板极少见，POX（+），考虑为AML-M_2_。骨髓活检+免疫组化（图3）：造血容量70 VOL％，骨髓增生较活跃，骨小梁间原始幼稚细胞弥散分布（30％），全片偶见巨核细胞，纤维组织灶性轻度增生。Gomori染色：MF-1级。免疫组化：CD3（−），CD20（−），CD34（+），MPO（+），CD33（+），CD117（+），CD99（+），CD10（−），TdT部分（+），Ki67（40％）。流式细胞术：原始细胞分布区域可见异常细胞群体，约占有核细胞的25.5％，主要表达HLA-DR、CD13、CD34、CD36、CD58、CD117、CD123、TdT，部分表达CD7，考虑AML。43种白血病融合基因阴性。染色体核型46，XY[20]。

**图1:**
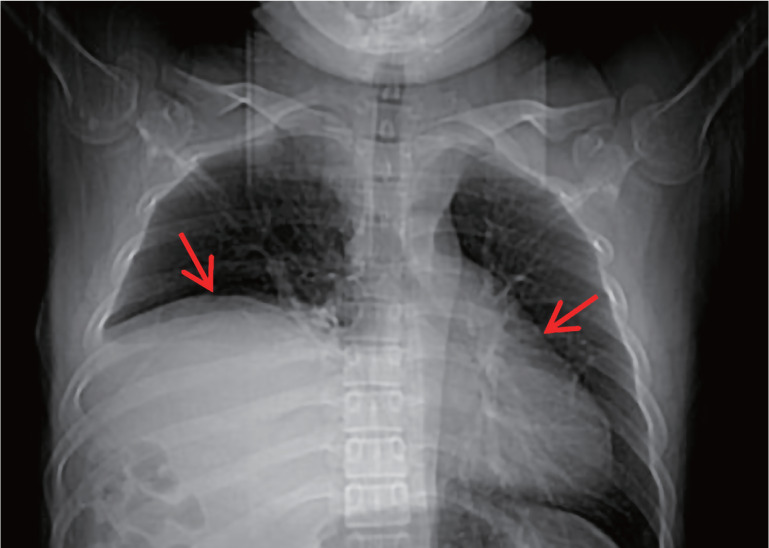
患者CT检查示右膈膨升伴纵隔及心脏左移

**图2:**
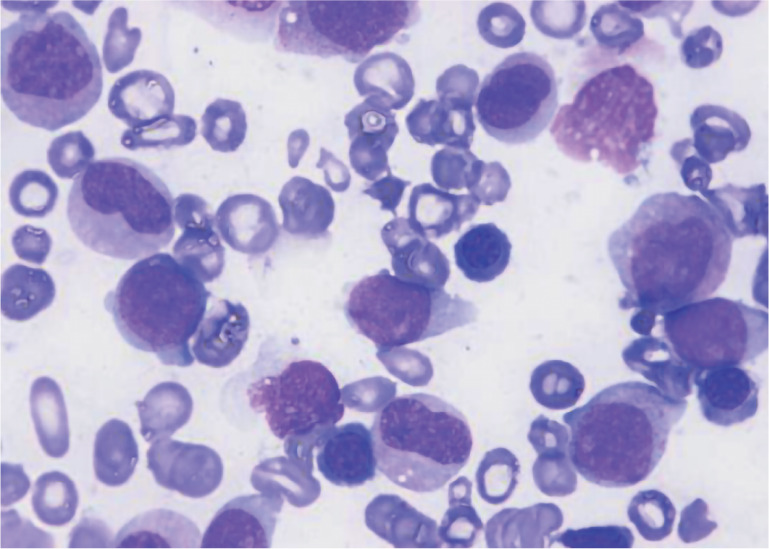
骨髓穿刺涂片细胞形态（×1000）

**图3:**
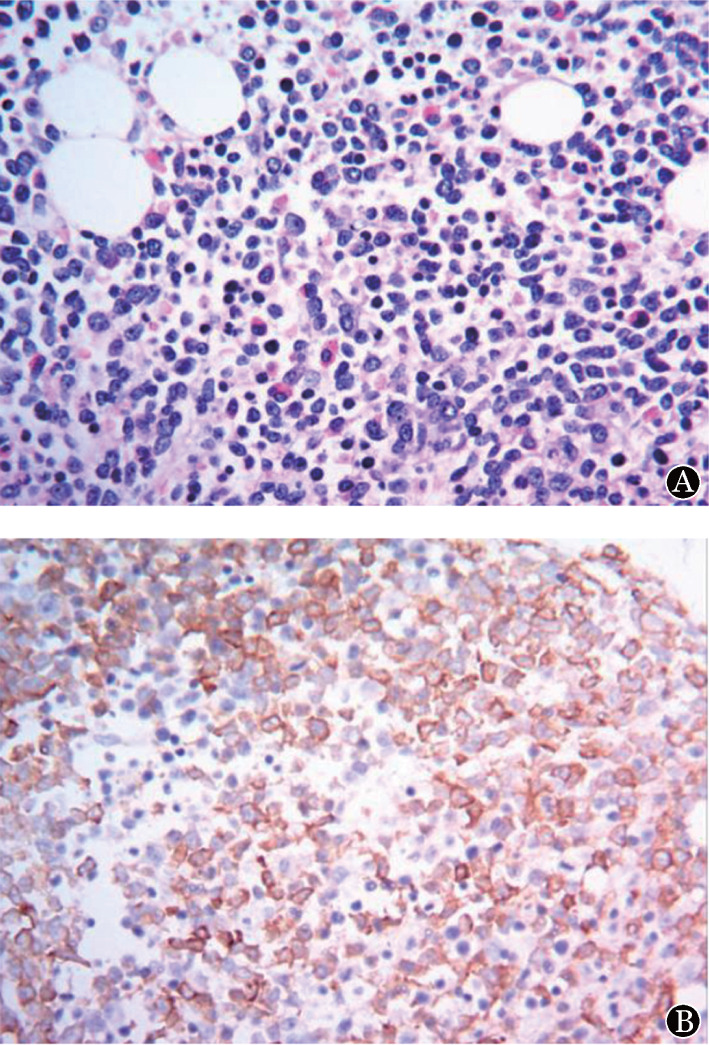
骨髓活组织病理检查（A，HE染色，×400）和CD33免疫组化（B，×400）

家系中共有5名成员存在血液系统疾病（图4）。患者母亲（Ⅱ_3_）为该家系先证者，于1990年产前检查检发现“血小板减低”，此后反复全身皮肤瘀斑30余年，2013年人流手术时输注血小板治疗，48岁死于“FPD-MDS/AML”。患者（Ⅲ2）2006年被诊断为急性单核细胞白血病，治疗1个疗程后因其父母自认治愈未再复查随诊，直至此次发病。外公（Ⅰ_1_）30多岁时曾反复发生自发性皮肤片状青紫，于武汉协和医院诊断为“AML”，55岁死于该病。舅舅（Ⅱ2）间断全身皮肤瘀斑10余年，2017年因家族史筛查血常规发现血小板减少，门诊随访血小板计数为（70～90）×10^9^/L；2017年患者表妹（Ⅲ_1_）因家族史筛查血常规亦提示血小板减少，门诊随访血小板计数（80～100）×10^9^/L，目前尚无其他显性临床症状。患者（Ⅲ_2_）及其母亲（Ⅱ_3_）、表妹（Ⅲ_1_）行血液肿瘤全外显子基因测序，均检出RUNX1基因第6号外显子错义突变c.562A>C，p.Thr188Pro，NM_001754，经验证均为胚系来源（表1）。家系成员Ⅱ_3_、Ⅲ_1_检测到RUNX1基因第9号外显子错义突变c.1415T>C, p.Leu472Pro, NM_001754，经验证为胚系来源。另外，先证者（Ⅱ_3_）检出NRAS、TET2、CEBPA体细胞突变。患者（Ⅲ_2_）检测到RUNX1、KIT体细胞突变。

**图4:**
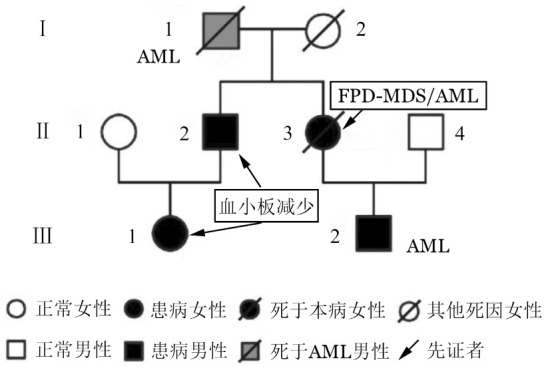
家族性血小板疾病（FPD）伴急性髓系白血病（AML）患者家系图 MDS：骨髓增生异常综合征

**表1:** 家族性血小板疾病（FPD）伴急性髓系白血病（AML）患者及2名家族成员的RUNX1基因二代测序结果

家族成员	基因	转录本	核苷酸改变	氨基酸改变	突变类型	突变频率（％）
患者母亲（Ⅱ_3_）^a^	RUNX1	NM_001754	c.562A>C	p.Thr188Pro	错义突变	34.19
	RUNX1	NM_001754	c.1415T>C	p.Leu472Pro	错义突变	43.34
	NRAS	NM_002524	c.179G>T	p.Gly60Val	错义突变	16.44
	CEBPA	NM_004364	c.611delC	p.Pro204ArgfsTer114	缺失突变	26.15
	CEBPA	NM_004364	c.68_69insC	p.His24AlafsTer84	插入突变	–
	TET2	NM_001127208	c.4121G>A	p.Cys1374Tyr	错义突变	19.42
患者表妹（Ⅲ_1_）	RUNX1	NM_001754	c.562A>C	p.Thr188Pro	错义突变	37.62
	RUNX1	NM_001754	c.1415T>C	p.Leu472Pro	错义突变	40.32
患者（Ⅲ_2_）	RUNX1	NM_001754	c.562A>C	p.Thr188Pro	错义突变	39.60
	RUNX1	NM_001754	c.485G>A	p.Arg162Lys	错义突变	43.70
	KIT	NM_000222.2	c.2447A>T	p.Asp816Val	错义突变	13.70

注：^a^：先证者

诊断为①FPD/AML伴胚系RUNX1单位点突变及获得性RUNX1、KIT突变；②横膈发育异常。2020年5月给予地西他滨联合HAG方案诱导化疗。化疗结束复查骨髓象：增生重度减低，原始细胞占8％，全片巨核细胞未见。6月25日给予阿扎胞苷联合Bcl-2抑制剂及达沙替尼再诱导，复查骨髓象：增生重度减低，原始细胞占16.5％。考虑为难治性AML，选择进行挽救性allo-HSCT。预处理：克拉屈滨5 mg·m^−2^·d^−1^，−11 d ～−9 d；阿糖胞苷2 g·m^−2^·d^−1^，−11 d ～−9 d；重组人粒细胞集落刺激因子5 µg·kg^−1^·d^−1^，−11 d ～−9 d；白消安3.2 mg·kg^−1^·d^−1^，−8 d ～−6 d；环磷酰胺1.8 g·m^−2^·d^−1^，−5 d～−4 d；ATG 2 mg·kg^−1^·d^−1^，−5 d ～−2 d；司莫司汀胶囊250 mg/m^2^，−2 d。2020年9月15日回输HLA全相合无关供者外周血造血干细胞，顺利植入，无明显并发症。复查AA诱导血小板最大聚集率为83.1％，较移植前明显改善。

## 讨论及文献复习

1978年Luddy等[1]报道了一个家系中3个兄弟姐妹死于骨髓增殖性疾病，既往有出血性疾病和血小板减少病史。1999年Song等[2]首次在6个家系中描述了胚系RUNX1基因的杂合突变，每个家系携带不同的突变位点。目前已报道130多个家系共包含104个不同的胚系RUNX1突变，每个突变对一个家系来说都是唯一的[3]。目前，国内仅报道1例RUNX1胚系突变的FPD/AML儿童患者[4]。

RUNX1是核心结合因子转录复合体的α亚基，有RUNX1a、RUNX1b和RUNX1c三种mRNA，均含RHD（runt-homology domain）同源结构域，RHD可特异性识别增强子核心区域从而介导RUNX1与DNA结合，调节许多与造血有关的基因表达，特别是巨核细胞的生成。大约10％的AML患者存在RUNX1突变[5]，在伴有RUNX1突变的AML患者中，30％为胚系突变[6]。

RUNX1胚系突变损害巨核细胞的分化和成熟，导致血小板的功能和数量缺陷，临床表现为出血、轻中度血小板减少、血小板形态正常，30％～40％的患者转化为MDS或AML[7]，少数转化为ALL、MPN等，还可合并实体肿瘤、湿疹和关节炎[3]。血小板功能缺陷包括血小板聚集功能降低，如δ-颗粒分泌缺陷导致的阿司匹林样血小板聚集功能异常[8]。本例患者初诊时AA诱导血小板最大聚集率为16.5％（参考值40.0％～80.0％），而HSCT后该结果为83.1％，明显改善。

胚系RUNX1突变的携带者不足以发展为白血病，获得额外的突变是FPD/AML疾病进展的关键。有研究显示RUNX1的体细胞突变是胚系RUNX1突变中最常见的获得性突变[9]，这表明双等位基因RUNX1突变足以导致恶性转化。2014年日本学者研究发现胚系RUNX1突变的FPD/AML在恶变过程中，体细胞CDC25C突变和GATA2突变分别是序贯白血病前期事件和继发性突变事件的重要因素[10]。RUNX1突变的类型（单倍体不足与显性负效应）或突变的位置影响白血病的转化倾向[11]，大多数RUNX1突变导致单倍体不足[2]，而一些突变以显性负性方式起作用，具有显性负效应的突变进展为白血病的概率更高[12]。通常错义突变和无义突变产生显性——负性作用，而移码突变和大段缺失导致单倍体功能不足。研究表明，RUNX1活性减半会导致原始红细胞生成、巨核细胞生成和血小板形成缺陷；而RUNX1活性完全丧失则导致基因组不稳定性增加，致使粒单核细胞扩增[11]。胚系RUNX1的错义突变体接近于完全功能丧失，而RUNX1缺失导致的RUNX1活性减半，表现为单倍体不足。多数情况下，单倍体不足导致血小板减少，而基因缺失更易导致白血病。本例患者为RUNX1基因第6号外显子c.562A>C（p.Thr188Pro）错义突变，突变位置为RHD，30岁进展为AML。从这个家系观察到，随着胚系基因的遗传，恶性转化的年龄越来越低。也有学者观察到同样现象[13]，因此，对家系最年轻的一代进行密切随访尤为重要。另外，本例患者横膈发育异常是否与胚系基因突变相关，目前未无相关文献报道。因家系成员Ⅰ_1_发病时为AML高发年龄，未行胚系验证，回顾家族史，考虑为FPD/AML可能性大。

对于FPD/MM的研究分析显示，在小于50岁的无症状胚系RUNX1突变携带者中，67％存在克隆性造血[14]，这些发现表明，在FPD/MM中克隆性偏斜的造血往往先于显性MDS/AML的发展[14]。至于克隆造血是否可以预测白血病转化的风险，尚需深入研究。

目前尚无伴RUNX1胚系突变FPD/MM的临床指南，可参照非胚系突变AML治疗方案。FPD/MM确诊后进行造血干细胞移植能否延长生存期目前无循证学依据。2021年美国国立卫生研究院（NIH）提出如下临床诊疗建议[15]：FPD/MM被认为不是单靠化疗就能治愈的疾病，几乎总是需要进行造血干细胞移植，allo-HSCT可以考虑用于有早期恶性肿瘤征兆的符合移植条件的患者。2020年Lachowiez等[16]报道了7例伴RUNX1胚系突变的血液系统恶性肿瘤患者，其中6例接受allo-HSCT；4例AML患者移植后3例分别死于复发、腺病毒感染和不明原因，中位生存期2.9年；另外2例MDS患者接受了清髓性无关供者造血干细胞移植，中位随访3.6年，处于缓解期；1例ALL患者准备接受造血干细胞移植。有限的资料显示此类患者进行可能受益于造血干细胞移植，但病例数太少，结果可能存在偏差。

本家系突变位点未见报道。根据美国医学遗传学与基因组学学会（ACMG）指南[17]，此突变位点可能是遗传疾病的致病性位点。伴RUNX1体细胞突变的AML患者预后不良，双等位基因RUNX1突变与更差的预后相关[5],[18]。本例患者除伴胚系来源的RUNX1单位点突变，还有体细胞RUNX1、KIT突变，且对常规化疗反应差，属高危难治性AML。有学者用FPD/AML家族患者的诱导多能干细胞（FPD-IPSC）进行研究，在FPD-IPSC中发现巨核细胞分化缺陷[19]，这种巨核细胞表型能够通过野生型RUNX1基因靶向纠正RUNX1突变来进行挽救。RUNX1突变的基因纠正有望为FPD/AML患者建立一种新的治疗策略。

临床医生怀疑胚系突变时，有必要对伴RUNX1突变的髓系肿瘤进行胚系验证，尤其是RUNX1双突变的患者。allo-HSCT仍是目前唯一治愈伴RUNX1胚系突变髓系肿瘤的方法。应避免选择RUNX1胚系突变携带者作为造血干细胞供者。RUNX1突变的外显性可能是不完全的，进展为骨髓恶性肿瘤的风险难以估计，定期随访尤为重要。
